# Struggling over recognition: Honneth, political resistance, and violence

**DOI:** 10.3389/fsoc.2025.1693721

**Published:** 2026-01-14

**Authors:** Renante D. Pilapil

**Affiliations:** Department of Philosophy, Ateneo de Davao University, Davao City, Philippines

**Keywords:** just cause, misrecognition, proportionality, social conflict, violence

## Abstract

This paper explores the extent to which recognition struggles can be considered as legitimate by looking into both their ends and means. It probes into the conditions why such political resistance is waged and whether or not violence is warranted as a necessary means to achieve legitimate political objectives. The paper argues that to make struggles for recognition legitimate, they should be motivated by a just cause such as experiences of oppression as is the case of misrecognition. To prevent accusations that such experiences of injustice are subjective, they have to pass the test of the publicity criterion. Meanwhile, although recognition struggles can become violent particularly in the context of political resistance, they need not be. Violence can be resorted to as a last resort but it has to be regulated by the principle of proportionality, meaning, the use of violence does not lead to more injustices. In the final analysis, violence has to be kept at the minimum because what defines a social protest or political resistance is not the use of violence but restraint and control.

## 1 Introduction

As a “vital human need,” recognition takes multiple forms depending on the sphere of social interaction (Taylor, 1995, p. 26; Honneth, 1995). In intimate relationships, human beings need love; in legal relations, they need respect; and in social relationships, they need esteem. These are respectively important for developing and maintaining the corresponding practical self-relations of self-confidence, self-respect, and self-esteem. Through acts of social recognition, individuals will achieve self-realization “defined as the process of realizing…one's self-chosen life goals” without coercion, that is, free not only from external but also from internal barriers (Honneth, 1995, p. 174). Meanwhile, non-recognition or misrecognition, as in the case of not being loved, being disrespected, and disesteemed, “can inflict a grievous wound, saddling its victims with a crippling self-hatred” (Taylor, 1995, p. 26). In the end, the ethical-psychological effects of misrecognition will contribute to individuals' not being able to function as individuated autonomous agents, thereby, losing their chance to attain self-realization.

Misrecognition does not only produce consequences affecting the personal life of individuals but also politically. According to Honneth (1995), the moral experience of disrespect can become the motivational impetus for political resistance. Victims of misrecognition can band together and engage in a social struggle or political resistance in order to demand justice. Here, Honneth links the distorted processes of individual experience of misrecognition with the processes of collective oppression and marginalization of groups (Benhabib, 2002, p. 51).

Just as any political resistance, the threat or inevitability of violence arises. Militaristic groups driven by violence will use the same argument in their struggles, whatever their political goals are. Renault (2011, p. 246) rightly points out that struggles for recognition may not be necessarily motivated by obtaining positive recognition but rather by violent destruction of the agents of misrecognition to let them pay for the injustice (justified or not) which they committed. In the last decades, events such as ethnic cleansing, religious fundamentalism, secession movements, terrorist attacks, among other events have blatantly demonstrated the escalation of violence in struggles for recognition. One such example is the ethnoreligious conflict between Christian Pana Dalits and Hindu Kandhas Adivasis in Kandhamal, India (Hota, 2023). Largely tied to Hindu ethnonationalism and the claim for recognition of Christian Dalits, the tension has been very violent as concretely shown in riots, burning of churches and houses, and murder.

This paper explores the extent to which recognition struggles can be considered as legitimate by looking into both their ends and means. It probes into the conditions why such political resistance is waged and whether or not violence is warranted as an instrument to achieve legitimate political objectives. The paper argues that for struggles for recognition to be legitimate, they should be motivated by a just cause such as the experience of oppression as is the case of misrecognition. To prevent such experiences of injustice to be accused of being subjective, they have to pass the publicity criterion. Meanwhile, although violence is an open possibility in recognition struggles typical of political resistance, they need not be. Violence can be resorted to as last resort but it has to be regulated by the principle of proportionality, meaning, the use of violence does not lead to more injustices. In the final analysis, violence has to be kept at the minimum because what defines a social protest or political resistance is not the use of violence but restraint and control.

The paper will proceed in six parts. The first section discusses how misrecognition can lead to political resistance, followed by the second section which shows the threat or inevitability of violence in recognition struggles. The third section discusses the principle of justice cause as standard for legitimate recognition struggles. This is followed in the fourth section with a discussion on violence as a political instrument. The fifth section will discuss the Moro rebellion in Mindanao, Philippines as a test case. The last section will present a brief conclusion.

## 2 Misrecognition and social conflicts

As early as the modern period, Jean-Jacques Rousseau in his work *Discourse on Inequality* and *On Social Contract* points out that human beings have the natural drive for *amour propre*—the desire “to have a position, to be part, to count for something” (OC 4, p. 421). That is to say, they need “to be esteemed, admired, or thought valuable” (Neuhouser, 2010, p. 22). In contemporary times, Taylor (1995, p. 26) similarly holds that due recognition is not only something we owe to people, but also it is a “vital human need.” Unlike Rousseau who implicates the desire for recognition to both human and social evils such as inequality, vice, competition, rivalry, and conflict (DI 184/OC 3, p. 189), Taylor highlights the impact of misrecognition or denial of due recognition to the internal life of the person. He says that misrecognition “can inflict a grievous wound, saddling its victims with a crippling self-hatred” (Taylor, 1995, p. 26).

Against this backdrop, Honneth rehabilitates the concept of recognition by tracing the latter to Hegel's philosophy of intersubjectivity and its naturalistic foundation in Mead. He argues that persons do not only desire recognition but they need multiple types of recognition depending on the sphere of social interaction (Honneth, 1995). In intimate relationship, persons need love; in legal relations they need respect; and in social relationships they need esteem through which the practical self-relations such as self-confidence, self-respect, and self-esteem are respectively developed. For Honneth (1995, p. 174), acts of social recognition are social preconditions for self-realization defined as the “process of realizing…one's self-chosen life-goals” without coercion. Conversely, experiences of misrecognition as in the case of disrespect can inflict wounds upon the self and cause humiliation—the feeling of being unwanted or unworthy in society, as if one's life possesses no significance or integrity of its own. The ethical-psychological impact of misrecognition can potentially hinder individuals from being able to fully function as individuated autonomous agents, thereby, losing their chance to attain self-realization (see also Butler, 1987; Brown, 1995; Allen, 2021; McQueen, 2014; Hörnsqvist, 2024).

According to Honneth, the consequences of misrecognition are not only personal but also political in that the moral experience of disrespect can become the motivational impetus for political resistance. Because of misrecognition, individuals can mobilize themselves and engage in a collective political struggle in the name of recognition of their moral and cultural identities. The struggles of various social movements waged by women, immigrants, laborers, LGBTQ+, and other minority groups exemplify this phenomenon. These groups are not only motivated by a fair share of economic resources but more importantly recognition of their unique identities.

The assumption here is that, because political resistance is essentially a group-led action, the individual experience of misrecognition can turn into a collective oppression or group marginalization (Benhabib, 2002). Individual injuries become collective injuries to self-confidence, self-respect, and self-esteem. To make this possible, Honneth (1995, p. 163) says that a “shared semantics” is required drawn from the victims' articulated stories of humiliation. The shared meanings will constitute the “intersubjective framework” through which the experience of suffering can be interpreted as affecting not only the individual but also others. The hope is that a “subcultural horizon of interpretation” will be eventually created within which fragmented and private experiences of disrespect become the “moral motives for a collective struggle for recognition” (Honneth, 1995, p. 164).

Linking the personal impact of misrecognition to collective political resistance or collective struggles for recognition is not an easy but a complex process. There is no automatic homology between individual and collective claims because there is no inherent coherence or unity of these claims owing to the possibility of conflict or tension. Individual interests do not necessarily coincide with collective interests. The meaning and interpretation of the experience of abuse, discrimination, and humiliation—being very personal—can differ from one person to another even as they share the same gender, race, ethnicity, or socio-economic status. Swanson (2005, p. 105) calls this the “politics of individual responses to injustices.” In the face of suffering or oppression, some deny their oppressive condition; others opt to legitimize their oppression as natural or socially beneficial; or, others actively engage in political resistance. And even as injustice has been recognized, “devising strategies for collective action and feeling able to act” are two different things. “Oppositional consciousness” does not necessarily translate into concrete political struggle (Mansbridge, 2001, p. 238–264).

Apart from these personal and existential conditions, the social, cultural and political environments can potentially limit political agency of the victims of recognition. In the absence of social movements through which the experience of disrespect is articulated and publicly manifested, it might be difficult for the victims to collectively organize themselves (Honneth, 1995, p. 260). One basic political challenge in the fight against injustice is not only the recruitment of political subjects who will join the cause but also the political and public support of other cause-oriented organized groups. Meanwhile, the state—having more material resources and monopoly of political power—can engage in “processes of institutionalized individualization” where it supports, promotes, and rewards individualistic actions to interrupt cooperative mobilization. This occurs when the state gives social and political rewards to individuals who take risks in competitive labor markets (Honneth, 2007, p. 89). Furthermore, the state employs practices of cultural exclusion that systematically withhold the linguistic and symbolic means necessary for the public expression of the victims' grievances as in the case of controlling the media and public education (Honneth, 2007, p. 89).

The idea that the experience of disrespect can become the motivational impetus for political resistance forms part of Honneth's broader idea of the “moral grammar of social conflicts.” Drawing from Parsons's (1951) framework of systems theory, Honneth says that social conflicts can be described from the perspective of an aspiration for social recognition. In the ideal case, the institutions of the law, economy, and family perform the double function of preserving the system and of enabling the satisfaction of the expectations of recognition (Honneth, 2012a, p. 8). Their norms or values form the basis of the members' duties, the fulfillment of which subsequently paves the way for the latter to acquire social recognition. For example, in modern legal systems, when individuals are gradually included through the ascription of “subjective” rights as citizens, they will learn to respect each one as free and equal persons. However, in the non-ideal case, conflict or tension arises when people are disadvantaged of their rightful claims or when these justified claims are unjustly reduced. These social conflicts take the form of micro confrontations in everyday life (person to person) to militant uprisings by entire collectivities. Their common cause is the moral indignation due to experiences of misrecognition. As such, these collective struggles for recognition are not arbitrary but rather justified on the basis of institutionally enshrined principles of recognition shared by all. As (Honneth, 2003, p. 186) puts it: a struggle for recognition proceeds via “a moral dialectic of the general and the particular…[in which] claims are made for a particular perspective (need, life-situation, contribution) that has not yet found appropriate consideration by appeal to a general recognition principle (love, law, achievement).”

However, Honneth points out that in the last part of the 20th century, political, cultural, and economic upheavals have shaken and significantly re-shaped the social struggles for recognition. In the context of legal relations, the struggle for equal rights of cultural minorities poses a challenge to the “active, empowering meaning of civil right” as “a symbol of reciprocal respect” in that it is now regarded “as instruments of securing personal performance” (Honneth, 2012a, p. 12). For example, a member of a particular identity group not accepted in a job being applied for could claim discrimination even as he/she is not qualified. Meanwhile, the fight for legal admission of immigrants, refugees, or illegal immigrants proves to be a more difficult, controversial case. Citizens of rich countries who regard fundamental human rights as a source of reciprocal recognition interpret the same principles as a “means of warding off what they feel as unacceptable claims” by immigrants (Honneth, 2012a, p. 12). In other words, the same language of human rights has been used to fuel anti-immigrant sentiments. In the end, Honneth reveals that in the past decades social conflict has been “barbarized” in that the struggle for recognition has lost its moral basis. Calling it a “brutalization of social conflicts,” Honneth (2012a, p. 17) says that members of the “underclass” groups battle for “public visibility or compensatory self-respect” where “idiosyncratic rules of recognition prevail.”

## 3 The threat or inevitability of violence

As forms of social conflicts, struggles for recognition produce a political consequence. Just as any political resistance that cannot resist using violence, recognition struggles grounded in moral-psychological premises could potentially lead to violence. Militaristic groups and malevolent groups, say ultranationalists and fundamentalists, whatever their political goals are, all of them are driven by violence, will use the same argument of social disrespect in their struggles. Renault (2010, p. 246) rightly points out that struggles for recognition may not be directly motivated by obtaining positive recognition but rather by violent destruction of the agents of misrecognition to let them pay for the injustice they had committed. Hence, struggles for recognition have the strong inclination toward legitimating what Ricouer (2005, p. 218, 246) calls individuals' “lust for power” and “fascination of violence.” The emphasis on militancy and conflict, he writes,

“only end[s] up as an indefinite demand, a kind of ‘bad infinity'…[such that] the temptation here is a new form of ‘unhappy consciousness,' as either an incurable sense of victimization or the indefatigable postulation of unattainable ideals.” (Ricouer, 2005, p. 218)

Historically, the fight against colonialism demonstrates this propensity toward violence. According to Fanon (1966, p. 63), “decolonization is always a violent event” as a response to the violent and brutal project of colonialism. The use of violence is not only a political but a clinical or existential necessity of colonized peoples/subjects. (Fanon, 1966, p. 94) writes:

“At the level of individuals, violence is a cleansing force. It frees the native from his inferiority complex and from his despair and inaction; it makes him fearless and restores his self-respect.”

Likewise, (Sartre, 1966, p. 21) declares:

“In the first days of the revolt you must kill: to shoot down a European is to kill two birds with one stone, to destroy an oppressor and the man he oppresses at the same time: there remain a dead man, and a free man.”

Both Fanon and Sartre audaciously endorse violence in anti-colonial resistance because of its cathartic and purifying effect. It offers the colonized peoples the road toward self-redemption, self-possession, and self-identity. By killing the colonizer, the native is reborn.

In the last decades, events such as ethnic cleansing, religious fundamentalism, secession movements, terrorist attacks, among others have blatantly demonstrated the escalation of violence in recognition struggles. Hota (2023), for example, provides an account of the ethnoreligious conflict between Christian Pana Dalits and Hindu Kandhas Adivasis in Kandhamal, India. Largely tied to Hindu ethnonationalism, on the one hand, and to the claim for recognition of Christian Dalits, on the other hand, the tension has been very violent as concretely shown in riots, burning of churches and houses, and murder. Meanwhile, the struggles for recognition waged by armed state non-actors (ANSA) illustrate another example of how these struggles can be violent. “Not integrated into formalized state institutions” and possessing “a certain degree of autonomy with regard to politics, military operations, resources, and infrastructure,” ANSAS are defined by their willingness and capability to use violence in pursuit of their objectives—political and/or economic (Hofmann and Schneckener, 2011, p. 604). As such, recognizing ANSAs (who are typically called terrorists) can be tricky in that doing so “can be conducive to both conflict transformation *and* escalation” as illustrated by the case of the Taliban in Afghanistan (Pfeifer et al., 2022, p. ix).

Honneth is not unaware of the possibility of the use of violence in recognition struggles. The question, however, is: How far can he exactly accommodate violence as an instrument to attain justified recognition? Faithful to his version of critical social theory, Honneth neither endorses nor denounces in advance the use of violence in recognition struggles. For example, when he presents the 2005 French suburban riots as motivated by a denial of respect on the part of the police and national security forces of the youth from immigrant backgrounds in poor parts of Paris, he certainly does not intend to provide a moral justification of gang-violence but rather an explanation of the justified motivations that produced them. Elsewhere, Honneth (1995, p. 163) explicitly writes:

“If one interprets social struggles from the perspective of moral experiences…there is no theoretical commitment in favor of either non-violent or violent resistance. Instead, at the level of description, it is left entirely open whether social groups employ material, symbolic, or passive force to publicly articulate and demand restitution for the disrespect and violation that they experience as being typical.”

His primary concern, Honneth (2007, p. 78) says, is how to empower the victims of injustice—the disrespected, humiliated, and ostracized—in such a way that they can articulate their experiences in the democratic public sphere, rather than live them “out in the counterculture of violence.” He aims to provide a “diagnosis” for “social pathologies” rather than an account of or a justification of right action. Instead of deliberately pursuing a normative evaluation of political actions, he aims for a reconstructive model of social theory that regards critique and conflict as immanent features of social life.

Bracketing questions about justifications can be interpreted as demonstrating the theoretical strength of Honneth's position. As one reviewer puts it, Honneth's view is “not of indecision but of methodological and philosophical consistency” in view of his broader project of treating recognition as a critical social category. Nevertheless, despite the veracity of this claim, this reading might be regarded as far from being complete especially when Honneth's project also aims to be a normative and moral account of social conflicts. Part of the methodology of critical (social) theory is the hermeneutical circle between the descriptive and the normative. In his earlier work, Honneth says that one cannot have a normative idea of what a just society is without already having some empirical observations about what is wrong with society. Conversely, to be able to make descriptive observations about what is wrong with society presupposes having already an idea of what justice is (Honneth, 2004, p. 390).

Contrary to the claim that Honneth does not engage in normative questions, Honneth (2012b) himself addresses the question of legitimacy as regards recognition struggles when he distinguishes between justified (morally imperative) and ideological forms of recognition. The latter specifically refers to how acts of recognition put subjects into structures of domination especially on the level of institutionally guaranteed recognition (as opposed to the level of intersubjective recognition). According to Honneth (2012b, p. 84), rules and practices of institutions could either *directly* or *indirectly* (as a side-effect) express patterns of recognition, thereby, contributing to how subjects (including their identities) are formed. The deliberate effort to create or discover new evaluative qualities that have to be given due recognition is crucial for questions on the ideological character of recognition. Against Althusser who regards all forms of ideology as irrational systems of belief, Honneth (2012b, p. 88) argues that “rational ideologies” exist when they “mobilize evaluative reasons with the power to rationally motivate their addresses to apply these reasons to themselves.” Subjects know and understand the reasons behind institutional acts of recognition (“historical space of reasons”) as opposed to blindly following them. What makes up an unjustified or irrational recognition? According to Honneth (2012b, p. 93), (institutional) acts of recognition are unjustified or irrational when they do not meet the material demands of those actions, when they do not do justice to “a new value quality in material forms,” that is to say, when they do not translate into actual transformation in the material life of recognition-receivers.

However, although such an account shows Honneth tackling questions about legitimacy, concerns about what forms and means the recognition struggles can take remain unclear. What happens when one group fights for a legitimate claim of justice but uses violent means? Will it make its legitimate claim illegitimate? Honneth may say that the means and forms of recognition struggles cannot be pre-determined but rather the situation depends on the assessment of the internal dynamics and specific contexts of these struggles. This “open” stance on the use of violence in recognition struggles begs the question as to whether or not the descriptive and the normative function of critical theory can be consistently held.

In this respect, I see the need to fill this lacuna in Honneth's account. I would like to explore the extent to which recognition struggles can be considered legitimate by looking into the ends justifying such political resistance and the use of violence in pursuit of these ends. To do this, I will draw upon the theory of just war most particularly the principle of just cause and principle of proportionality. Although political resistance is different from war in terms of methods, goal, and scope—the latter is more often large-scale and aims to overthrow government or conquer territories—both of them are forms of challenging power that utilize both violent and non-violent means. Generally, “resistance” refers to a “broad range of dissident activities, of varying scope and impact, which express opposition, and perhaps refusal to conform, to a dominant system of values, norms, rules, and practices” (Delmas, 2020, p. 17). Political resistance is different from everyday resistance in that it is collective, intentional, and public in nature, examples of which include street riots, civil disobedience, and revolutions. Also, it can be lawful or unlawful, violent or non-violent, civil or uncivil directed against a state or another agent (Bilginer, 2015; Delmas, 2020). Let me start with the issue on the ends of political resistance.

## 4 Just cause as end of recognition struggles

According to just war theory, war can be resorted to when it is done out of a just cause. Lackey (1989, p. 33) says that as early as Cicero in the first century B.C., a just cause has been understood as a cause fought for because of a wrong received from another party through humiliation, insult, violation of human rights, verbal disrespect, desecration of national or cultural symbols, among other things. In this sense, to fight for a just cause is a quest for moral reparation for what has been tarnished or lost—pride, honor, identity, and dignity—which are essential to being human and ought to be respected (Lackey, 1989, p. 34–35). In international relations, a just cause applies to situations when states are morally permitted to engage in war against another state in order to disarm an aggressor, deter future aggression, prevent lesser humanitarian crimes (McMahan, 2005). Some examples of just cause are national defense, defense of right, or self-determination (Walzer, 2006). In the case of political resistance, what can be considered as a just cause includes liberation from oppression or the achievement of political freedom (Arendt, 2006). As such, when a political resistance—whether lawful or unlawful—is made out of a just cause, then, it is a *principled* political resistance.

The same criteria for a just cause can be applied to struggles for recognition. This is the case particularly when institutional rules and norms violate or deny the normative expectations of political subjects upon the social order, central of which is the recognition of their needs and emotions, moral autonomy, and traits and abilities. When they are humiliated or disrespected, and excluded not only from their political community but also from the community of personhood, individuals and groups have the just cause to engage in struggles for recognition. When a cultural group is denied their right to determine their own political status, culture, and future, or when they seek reparation for the historical injustice they went through for many years, they have a just cause to engage in the struggle for recognition. Apart from these unjust institutional acts of misrecognition, it is also possible that at the level of intersubjective relationships individuals or groups may also experience misrecognition that can serve as just cause. This happens particularly when through gestures, expressions, or speech, the positive qualities of a person or a group is denied affirmation so that the addressee cannot identify with his or her own qualities, thereby, preventing him/her to achieve greater autonomy. Examples of which include personal attitudes like disdain, suspicion, or distrust against a person or group by virtue of his/her race, ethnicity, religion. Adulterating Levinas, these are instances of “allergy” toward the other. Indeed, a just cause is not only a political but more importantly a moral goal.

Nevertheless, determining a just cause especially in the case of recognition struggles is tricky. Populist, xenophobic, and right-wing movements who claim being disrespected or misrecognized may use the same reason of just cause and attempt to engage in recognition struggles. In recent years, these pseudo “social movements” have become very active in public life. They include white supremacist groups like the German skinheads, anti-Semitism groups, the Hindu nationalist movement Rashtriya Swyamsevak Sangh (RSS) in India, among others. The point here is that individuals and groups may struggle for recognition on the basis of perceived injustice or on unjust and evil grounds couched in the language of just cause (Senf, 2023; Zurn, 2023).

In this situation, the need for a publicity criterion in the struggle for recognition becomes necessary to prevent the latter from being prone to subjectivist interpretation or from being used as a pretext for malevolent purposes (Fraser, 2003; Pilapil, 2011). The publicity criterion is an important means through which legitimate and illegitimate demands for recognition are adjudicated. A claim for recognition is only considered justified when it is publicly articulated, verified, and defended through the process of discourse which involves an exchange of reasons, that is, through critical scrutiny, convincing those from whom recognition is demanded (Forst, 2010; Knieriem, 2024). When those from whom recognition is demanded do not see the injustice supposedly claimed by a group, or social movement, then, the demand for recognition may not be considered as legitimate.

The case of recognition struggles involving armed non-state actors (ANSAs) in situations of intrastate wars may prove to be more complex. To demand a publicity criterion for these groups who are labeled as “terrorists” in order to rationally test their recognition claims—whether these are a political cause or identity—may not work at all. Governments (most particularly from the West), international organizations, local populations and other groups—which can be collectively called as recognition-granters—are at a dilemma whether or not to engage in talks with them, whether they start to accommodate them or act in a hostile way against them. Also, granting them recognition will have important implications for conflict management and transformation or escalation (Pfeifer et al., 2022). Such was the case of the Taliban which took the reins of power in Afghanistan after the withdrawal of NATO troops as part of the controversial peace agreement in 2020. At some point, many actors had no choice but to engage with them for the sake of humanitarian aid for the population as well as in the fight against ISIS (Anderson, 2021).

Let me now proceed to the pertinent issue as to whether or not struggles for recognition motivated by a just cause can employ violent means in the pursuit of their supposedly legitimate socio-political objectives.

## 5 Violence and the principle of proportionality

Following the logic of political resistance, the use of violence is an *open* possibility if what is desired is a change or disruption of an existing political or social order that violates or denies the legitimate demands of political subjects. Violent political resistance can fall into the category of what Delmas (2020, p. 19) calls as “uncivil disobedience,” one basic feature of which is violence (aside from covertness, evasion, and offensiveness), as opposed to “civil disobedience” which is public, nonviolent, and respectful—both of which aims to achieve any of these goals namely system overhaul, harm prevention, retaliation, expression of discontent, among others. At the edge of uncivil disobedience is terrorism which tends to use indiscriminate violence and intentional killing both of civilians and military targets with no regard for their right to life and bodily integrity (Delmas, 2020, p. 198).

In a democratic polity, violence is regarded as different from authorized force. While the latter involves the legitimate use of state power to prevent, restrain, or punish those who have violated the law, the former lacks the legitimacy of constitutional and legal sanction (Yet, this is in no way means that the state does not employ violence). Honderich (1989, p. 151) defines violence as “a considerable use of force against persons or things, a use of force *prohibited by law* and directed to a change in the policies, personnel, or system of governments, and hence to changes in society” [my emphases]. Violence, then, involves employment of unlawful force and coercion, physical most particularly, with the intention of causing harm or damage to persons or properties. It ranges from smashing of windows, break ins, vandalism, arsons, and lootings, to assault, hijacking, maiming, and killing of persons.

Today, the term violence has broadened to include structural, symbolic, epistemic, psychosocial, and linguistic violence, encompassing a wide range of social injustice and inequalities (Coady, 2009, p. 244, 259). These descriptions of violence share in common the problem of “managing symbols and meanings in order to discount the voices and standpoints of some” which “has injurious effects on the discounted,” thereby, making them violent (Frazer and Hutchings, 2020, p. 3). Yet, one wonders whether these are really kinds of violence or merely a provocative motif or metaphorical use of violence, resulting to some sort of hurt or injury that is equivalent to suffering (Mardon and Richardson-Self, 2022). I will not attempt to resolve these conceptual problems here. For the purposes of this paper, I am more inclined to refer to a general description of violence as the use of destructive force—which could imply inflicting physical harm to people and properties, the most extreme of which is killing—in order to effect a change in policies, systems of governments, and hence of the society.

Just like any political resistance, recognition struggles can become violent because they disrupt and transform an existing order of reality that fails to give justice to the legitimate expectations of political subjects. The whole point of these struggles is not merely to vent out publicly the moral experiences or feelings of disrespected or humiliated groups and individuals. It is to demand the institutionalization of socially legitimate claims that are not yet considered legitimate through which the lost self-respect or self-esteem of the victims of misrecognition can be hopefully restored (Honderich, 1989, p. 311). Recognition struggles are not institutionally legitimate forms of resistance because they do not require political means or instruments approved by the state, say those used by civil society groups like holding protests, lobbying, among others. As such, according to Arendt (1970, p. 176; 1977, p. 142, 299), “violence may be ‘rational' and appropriate in certain crisis moments” including “liberation from oppression, the dramatization of grievances, and even attempts to gain a hearing.” In other words, violence is resorted to in order to achieve legitimate political objectives. Such is the case of a number of recognition struggles today particularly from decolonial or anti-colonial movements such as ethno-nationalistic separatist movements aiming for self-determination and recognition of identities.

However, to say that the use of violence in the pursuit of legitimate political resistance—that is, principled, based on just cause—as in the case of recognition struggles is an open possibility does not mean that violent means must be the first option. Violence should only be employed as a last resort, that is, only after all nonviolent (or peaceful) means—including deliberation, dialogue, negotiation, or other legal remedies—have been exhausted but are rendered futile. That is to say, there are no available means less dangerous, and more or less effective exist in order to achieve a just cause (Moody-Adams, 1999).

Concerns about the effectiveness of violent means in the political resistance in general, and in recognition struggles in particular are crucial. This is because it might be the case that the use of violence is not really necessary. Empirical evidence from the social sciences shows that violence is in fact counterproductive. The study of Chenoweth and Stephen (2011) shows that while many violence decolonization movements succeeded in the 1970s and 1980s, the success of violence resistance movements has since declined. In contrast, nonviolent movements have become increasingly successful since the 1950s and are twice as likely to succeed as violent campaigns. Nonviolent means have been employed in many political resistance movements such as political or economic boycott, civil disobedience, or symbolic protests. This is historically demonstrated by various nonviolent political resistance movements notably the civil disobedience movement in British India in the early 1930s.

Apart from this concern on the effectiveness of violent means in the pursuit of political objectives, violence is regarded as anti-democratic, against the rule of law, and against the basic duty to obey the law.

However, should violence be resorted to, it must be governed by the principle of proportionality. In just war theory, “the rule of proportionality states that a war cannot be just unless the evil that can reasonably expected to ensue from the war is less than the evil that can be reasonably expected to ensue if the war is not fought” (Lackey, 1989, p. 40). In other words, proportionality—which is both a legal and a moral principle—is primarily a balancing act between the necessity to use violence and the consequences it would produce. The use of violence should be proportionate to the context and ends. The point is that violent means should not result in worse injustice than when nonviolent means are employed. In that sense, violence becomes a form of lesser evil. For example, a group's use of violence is proportionate when it only harms those who sought to harm them or when it is essential for their collective self-defense against violence. It becomes excessive when it attacks the innocent in pursuit of horrendous ends.

This applies to situations of “supreme emergency.” In the context of war, this happens when there is the imminence of danger of death and military defeat as is the case of Hiroshima bombing where according to Landesman (2003), the use of the atomic bomb was necessary to end the war as quickly as possible and therefore saving many lives, whether American or Japanese. In political resistance, imminent danger can also take the form of unusual and horrifying danger in which justice, freedom, survival, and civilization are at risk. In other words, an unimaginable disaster to the political community is at hand as in the case of the experience of colonized peoples in Southeast Asia and Africa. Colonialism is a brutal project of “divide and conquer” decimating identities of nations and plundering their natural resources. In this situation, the sense of obligation and moral agency calls for the employment of violence, which if were not carried out, could lead to the continuation of injustice and oppression, loss of freedom, and even threatening survival.

For some critics, applying the principle of proportionality in the context of violence in political resistance such as recognition struggles and even in war may not be that straightforward. This is because it is difficult to calculate the consequences—probable or actual. According to Wilkinson (2000), there is no clear demarcation whether the demonstration of violence is final because of possible retaliatory attacks from the group attacked. Violence could spin out of control, that is, violence begets further violence. As Arendt (1970) puts it, violence can easily slide into atrocity and terror. This is particularly evident in ethnoreligious conflicts where the violent attack of one ethnic group against another ethnic group leads to retaliatory violent attacks from the latter. Such was what happened in Mindanao War where the attack of Muslim rebels against Christians led to the formation of armed Christian groups called the *Ilaga* (rodents) who were mainly motivated by attacking Muslim, either civilians or combatants. Secondly, the basis for weighing the cost of damages say between the relative cost of human lives as compared to ravaged building or a cultural artifact may not always be available. In other words, the weighing of the cost is complicated by the possible conflict of values of individuals who are calculating the damages.

To address these concerns, it might be useful to keep in mind that balancing is not simply premised on maximizing overall utility. When balancing one does not simply apply some objective or predetermined mechanism that can guide decision-making. Rather, as Legg (2012) puts it, “it is about determining which norm or reasons should guide the decision in a given case.” As a form of practical reasoning, balancing involves finding norms or reasons for choosing action A over B, or the other way around, and determining which among them must become the basis for a decision. Applied to the context of the use of violence, the principle of proportionality means finding the reasons for choosing violence as a political instrument over non-violent means in order to achieve a just cause. It is about rationally determining whether or not there are legitimate conditions that warrant the use of violence. This reasoning implies that, following the principle of proportionality, even as violence maybe permitted in exceptional circumstances, it should be kept at the minimum. As such, extreme violence like terrorist violence motivated by indiscriminate violence and intentional killing of civilians must not be permitted in struggles for recognition. As Walzer (2006, p. 205) puts it: “The mark of a revolutionary struggle against oppression…is not this incapacitating rage and random violence, but restraint and control.” Let me briefly discuss the Moro struggle in Mindanao, Philippines as a test case.

## 6 The Moro struggle in Mindanao, Philippines

Muslim resistance in Mindanao, Philippines began in the early 16th century when the Spaniards colonized the Philippines. While most parts of Luzon and the Visayas, two of the three main groups of Philippine islands, Mindanao remained free from Spanish rule even if the latter gained foothold in northern and eastern portions of Mindanao as well as in the Zamboanga peninsula (Majul, 1988). Part of the reason for this was the Moros had their own organized political system called the sultanate allowing them to successfully resist Spanish invasion. When the Philippines was transferred to the Americans through the Treaty of Paris at the end of the Spanish-American War in 1898, things began to change dramatically. The Philippine Commission, put up by the US colonial government, implemented the Regalian doctrine which granted the Philippine state the sole authority to grant land to its citizens, requiring them to register their ownership through land titles and setting limits on the land size allowed for ownership. The measure did not sit well with the Moros who believe that the right to hold land was based on the right to the produce of the land. That was the start of the Moro's dispossession of their lands, igniting pockets of resistance within the Mindanao island.

However, it was the American's transmigration policies in the 1920s that most effectively diluted the Moro's control over Mindanao when a mass of Christian Filipinos in the north, offered with subsidies, free land titles, animals and other equipment, migrated to Mindanao and formed Christian enclaves in Moro areas. Christian population rapidly increased in Mindanao resulting to their eventual minoritization and dispossession of their ancestral lands. At this point, it is important to note that despite their being unjustly treated, the Moros did not originally resort to an armed violent struggle. The turning point came in 1968 (more than 20 years after Philippine Independence) when members of the Philippine Armed Forces summarily executed about thirty young Muslim recruits who refused to carry out a secret mission to invade Sabah, Malaysia (Majul, 1988). Dubbed as the Jabidah Massacre, the incident pushed the Muslims, now the minority, to form private armies that launched retaliatory attacks against government military troops and Christian civilians. Supported by a mass base, Moro rebel groups like the Moro National Liberation Front (MNLF) and the Moro Islamic Liberation Front (MILF; a breakaway of MNLF) became formidable forces that sought to advance the Moro ideals of re-acquisition of ancestral lands and creation of a *Bangsamoro* Republic in Mindanao through armed violent revolution. In 2019, after countless efforts to achieve peace, the conflict finally found its conclusion with the establishment of the Bangsamoro Autonomous Region for Muslim Mindanao (BARMM).

More than the economic or material dimension, the Mindanao War was fueled by the moral-psychological impact, specifically the experience of disrespect and humiliation of Muslims brought about by marginalization and minoritization. The experience of disrespect—long and deep—is quite evident in their articulated narratives. From the period of Spanish and American colonization to the succeeding Philippine governments, they have never been treated worthy of equal respect as a people with their own identity and autonomy. They had been sold to the Americans; their lands forcefully grabbed; their cultural and political practices destroyed; their brothers massacred; and most aspects of their culture deemed “other” and “uncivilized” compared to Christian Filipinos. Muslims are perceived as terrorists, violent, devious, traitors, among other labels which the Manila-based media irresponsibly promote. Oftentimes, they are treated with suspicion, distrust, and social disdain especially by Christian Filipinos in the north. It is not improbable to say that these stifling conditions have produced a negative impact on the Muslims' sense of self-respect and self-esteem. Such moral suffering brought about by oppression, subjugation, and marginalization is crucial because, as Honneth points out, it can serve as the motivational impetus for political resistance. In other words, the historical injustice constitutes a just cause to engage in legitimate political struggles for recognition.

Nevertheless, while the Moro struggle is justified in view of its end, the use of violence is more nuanced. The military option—which could be violent—targeting government forces may have proved to be effective in forcing the Philippine government to take the issue of Moro struggle and their demands with more seriousness. For a long time, their demands—both political and moral—had fallen on deaf ears as if they are not a legitimate group to be listened to. In that sense, the Moro rebels are justified to engage in violent armed struggle. Yet, the same thing cannot be said in their use of terrorist violence including the burning of houses in Christian communities or bombing of a Catholic Cathedral and other churches, an international airport, shopping malls and commuter trains. There was no condition of systematic violation of human rights of the Muslims since they still enjoy the same basic human rights as their Christian brothers and sisters. Muslim children can go to school. Muslims can practice their religion freely. They can vote and participate in the elections, though it cannot be denied that discrimination took place. Hence, given these more or less favorable conditions, there is no overwhelming righteousness for the Muslims to resort to terrorist violence.

## 7 Conclusion

As argued by Honneth, misrecognition can become a motivational impetus for political resistance where the use of violence remains a threat or inevitable. Violence is an open possibility in these recognition struggles primarily because they disrupt and transform an existing reality requiring means or instruments not approved by the state. However, while recognition struggles can become violent, they need not be. Violence as a political instrument should only be resorted to after all nonviolent means have been explored and must be kept at the minimum.

In the final analysis, what can be said is that although the struggle for recognition is politically and morally powerful it is also highly dangerous. The worry is that pushed to the extreme the idea of struggle for recognition potentially suggests that the primary condition of people's relationship is one of constant conflict anchored in their desire for recognition, or in the Hobbesian language the desire for self-preservation. It tends to neglect and downplay the basic concern for what (Ricoeur, 2005, p. 246) calls “state of peace,” which is what we urgently need in our troubled world today. To say so does not mean that what should be aimed at is only the condition of eternal peace in which no conflicts occur at all. The challenge is how to make our societies less violent and less destructive than they are now. With the escalation of different forms of violence that afflict different parts of the world, the greater moral and political demand is how to minimize if not prevent rampant violence from happening. After all, it is not violence that defines the political (including political resistance and revolution). As Arendt (2005, p. 117) puts it, the real “meaning of politics” only “comes to the fore where people are with others and neither for nor against them—that is, in sheer human togetherness.”

## References

[B1] Allen A. (2021). “Recognizing ambivalence: Honneth, Butler, and philosophical anthropology,” in Recognition and Ambivalence, eds. H. Ikäheimo, K. Lepold, and T. Stahl (New York, NY: Columbia University Press), 99–128.

[B2] Anderson S. R. (2021). History and the Recognition of the Taliban. Available online at: https://www.lawfareblog.com/history-and-recognition-taliban (Accessed November 21, 2021).

[B3] Arendt H. (1970). On Violence. New York, NY; London: Harcourt, Inc.

[B4] Arendt H. (1977). On Revolution. New York, NY: Penguin.

[B5] Arendt H. (2005). The Human Condition. Chicago, IL: Chicago University Press.

[B6] Arendt H. (2006). Between Past and Future. London: Penguin.

[B7] Benhabib S. (2002). The Claims of Culture: Equality and Diversity in the Global Era. Princeton, NJ: Princeton University Press. doi: 10.1515/9780691186542

[B8] Bilginer O. (2015). Theories of political resistance: the making of resistance from the sixteenth century to the present (Ph.D. dissertation). University at Albany, State University of New York, Albany, NY, United States.

[B9] Brown W. (1995). States of Injury: Power and Freedom in Late Modernity. Princeton, NJ: Princeton University Press. doi: 10.1515/9780691201399

[B10] Butler J. (1987). Subjects of Desire: Hegelian Reflections in Twentieth-Century France. New York, NY: Columbia University Press.

[B11] Chenoweth E. Stephen M. (2011). Why Civil Resistance Works: The Strategic Logic of Nonviolent Conflict. New York, NY: Columbia University Press.

[B12] Coady C. A. J. (2009). “The idea of violence,” in Violence: A Philosophical Anthology, ed. V. Bufacchi (London: Palgrave Macmillan), 244–259.

[B13] Delmas C. (2020). Uncivil disobedience. Nomos 62, 9–44. doi: 10.18574/nyu/9781479848003.003.0006

[B14] Fanon F. (1966). The Wretched of the Earth. New York, NY: Grove Press.

[B15] Forst R. (2010). The justification of human rights and the basic right to justification: a reflexive approach. Ethics 120, 711–740. doi: 10.1086/653434

[B16] Fraser N. (2003). “Social justice in the age of identity politics: redistribution, recognition, and participation,” in Redistribution or Recognition: A Politico-Philosophical Exchange (London; New York, NY: Verso), 7–111.

[B17] Frazer E. Hutchings K. (2020). Violence and Political Theory. Cambridge: Polity Press.

[B18] Hofmann C. Schneckener U. (2011). Engaging non-state armed actors in state- and peace-building: options and strategies. Int. Rev. Red Cross 93, 603–621. doi: 10.1017/S1816383112000148

[B19] Honderich T. (1989). Violence for Equality: Inquiries in Political Philosophy. London: Routledge.

[B20] Honneth A. (1995). The Struggle for Recognition: The Moral Grammar of Social Conflicts. Transl. by J. Anderson. Cambridge: Polity Press.

[B21] Honneth A. (2003). “Redistribution as recognition: a response to Nancy Fraser,” in Redistribution or Recognition? A Political-Philosophical Exchange, eds. N. Fraser, and A. Honneth (London: Verso), 110–197.

[B22] Honneth A. (2004). From struggles for recognition to a plural concept of justice: an interview with Axel Honneth. Acta Sociol. 47, 380–391. doi: 10.1177/0001699304048674

[B23] Honneth A. (2007). Disrespect: The Normative Foundations of Critical Theory. Cambridge: Polity Press.

[B24] Honneth A. (2012a). Brutalization of the social conflict: struggles for recognition in the early 21st century. Distinktion 13, 5–19. doi: 10.1080/1600910X.2012.648736

[B25] Honneth A. (2012b). The I in the We: Studies in the Theory of Recognition. Cambridge: Polity Press.

[B26] Hörnsqvist M. (2024). Recognition of struggle: transcending the oppressive dynamics of desire. Constellation 31, 414–427. doi: 10.1111/1467-8675.12711

[B27] Hota P. (2023). The Violence of Recognition: Adivasi Indigeneity and Anti-Dalitness in India. Philadelphia, PA: University of Pennsylvania Press. doi: 10.2307/jj.362397

[B28] Knieriem M. (2024). The limits of recognition. Inquiry 1–36. doi: 10.1080/0020174X.2024.2338822

[B29] Lackey D. (1989). The Ethics of War and Peace. Hoboken, NJ: Prentice Hall.

[B30] Landesman C. (2003). Rawls on Hiroshima: an inquiry into the morality of the use of atomic weapons in August 1945. Philos. Forum 34, 21–38. doi: 10.1111/1467-9191.00122

[B31] Legg A. (2012). The Margin of Appreciation in International Human Rights Law: Deference and Proportionality. Oxford: Oxford University Press. doi: 10.1093/acprof:oso/9780199650453.001.0001

[B32] Majul C. (1988). The Moro struggle in the Philippines. Third World Q. 10, 1335–158. doi: 10.1080/01436598808420087

[B33] Mansbridge J. (2001). “Complicating oppositional consciousness,” in Oppositional Consciousness: The Subjective Roots of Social Protest, eds. J. Mansbridge, and A. Morris (Chicago, IL: Chicago University Press), 238–264. doi: 10.7208/chicago/9780226225784.001.0001

[B34] Mardon G. Richardson-Self L. (2022). Stuck in suffering: philosophical exploration of violence. Aust. Fem. Law J. 48, 113–136. doi: 10.1080/13200968.2022.2088947

[B35] McMahan J. (2005). Just cause for war. Ethics Int. Aff. 19, 1–21. doi: 10.1111/j.1747-7093.2005.tb00551.x

[B36] McQueen P. (2014). Subjectivity, Gender and the Struggle for Recognition. London: Palgrave Macmillan. doi: 10.1057/9781137425997

[B37] Moody-Adams M. (1999). The idea of moral progress. Metaphilosophy 30, 168–185. doi: 10.1111/1467-9973.0012011357779

[B38] Neuhouser F. (2010). “Rousseau and the Human Drive for Recognition (Amour Propre),” in The Philosophy of Recognition: Historical and Contemporary Perspectives, eds. H. C. am Busch and C. Zurn (Plymouth: Lexington Books), 21–46.

[B39] Parsons T. (1951). The Social System. New York, NY: Free Press.

[B40] Pfeifer H. Geis A. Clement M. (2022). The Politics of Recognition, Armed Non-State Actors, and Conflict Transformation. Frankfurt am Main: Peace Research Institute Frankfurt.

[B41] Pilapil R. (2011). Psychologization of injustice: on Axel Honneth's theory of recognitive justice. Ethical Perspect. 18, 79–106. doi: 10.2143/EP.18.1.2066214

[B42] Renault E. (2010). “Taking the inheritance of critical theory: saving Marx by recognition,” in The Philosophy of Recognition: Historical and Contemporary Perspectives (Plymouth: Lexington Books), 241–256.

[B43] Renault E. (2011). “Taking on the inheritance of critical theory: saving Marx by recognition,” in The Philosophy of Recognition: Historical and Contemporary Perspectives, eds. H.-C. Schmidt am Busch, and C. F. Zurn (Lexington, MA: Lexington Press), 223–240.

[B44] Ricoeur P. (2005). The Course of Recognition. Transl. by D. Pelauer. Cambridge, MA: Harvard University Press.

[B45] Ricouer P. (2005). The Course of Recognition, Transl. by D. Pelauer. Cambridge, MA: Harvard University Press.

[B46] Sartre J. P. (1966). “Preface,” in The Wretched of the Earth, ed. F. Fanon (New York, NY: Grove Press). 39488397

[B47] Senf C. (2023). Occupying a square? Recognition theory of social movements in the age of wealth-induced political inequality (Ph.D. dissertation). University of Bergen, Bergen, Norway.

[B48] Swanson J. (2005). Recognition and redistribution: rethinking culture and the economic. Theory Cult. Soc. 22, 87–118. doi: 10.1177/0263276405054992

[B49] Taylor C. (1995). “The politics of recognition,” in Multiculturalism: Examining the Politics of Recognition, ed. by A. Gutmann (Princeton, NJ: Princeton University Press), 149–164.

[B50] Walzer M. (2006). Just and Unjust Wars: A Moral Argument with Historical Illustration. New York, NY: Basic Books.

[B51] Wilkinson P. (2000). Terrorism Versus Democracy: The Liberal State Response. London: Frank Cass Publishers.

[B52] Zurn C. (2023). “Populism, polarization, and misrecognition,” in The Theory and Practice of Recognition, eds. O. Hirvonen, and H. J. Koskenin (New York, NY: Routledge), 131–149. doi: 10.4324/9781003259978-10

